# The smart home, a true home? How new technologies disrupt the experience of home for older persons

**DOI:** 10.1007/s10209-024-01114-1

**Published:** 2024-05-04

**Authors:** Nadine Andrea Felber, Hamed Alavi, Elena Mugellini, Tenzin Wangmo

**Affiliations:** 1 Institute of Biomedical Ethics, University of Basel, Bernoullistrasse 28, 4056 Basel, Switzerland; 2 Digital Interactions Lab, Institute of Informatics, University of Amsterdam, Amsterdam, Netherlands; 3 HumanTech - Technology for Human Wellbeing Institute, Haute École Spécialisée de Suisse Occidentale, Delémont, Switzerland

**Keywords:** Smart home, Health technologies, Older persons, Aging in place, Technology design

## Abstract

Smart home technologies (SHTs) can support older persons to age in place. However, adoption of SHTs remains low among this population. A reason for this is that they are not accustomed to having a home that is technologically enhanced. In this article, we focus on the older persons’ lived experience of “home” and show how SHTs potentially disrupt it. In consulting the currently available literature, both theoretical and empirical, we propose and use the concept of somatic capability assessment (SCA) in the discussion surrounding the design of SHTs for older persons. First, we propose SCA as a concept to grasp how humans take decisions while relying on their physical body, undisturbed by suggestions from technologies. Furthermore, we show that SCA functions best in a familiar and private environment—the home. SHTs have the potential to make the home seem unfamiliar and exposed, precisely through added data and the resulting suggestions, as we show through related empirical studies. Thus, SHTs hold an increased disruptive potential for older persons at home. By introducing SCA into the discussion of SHTs for older persons, and thus paying attention on how SHTs potentially disrupt the experience of home, further advances the ethical discussion on the adequate use and design of technologies in daily life, especially for the group of older persons. Our analysis offers important insights for the design and implementation processes of SHTs for older persons.

## Introduction

An important priority for older persons is the ability to stay in their own home [1]. Home, in this context, denotes a place that someone is familiar with, has control over and is sheltered from the unwanted outside [2]. In old age, when both physical and mental capabilities decline and ailments become more frequent [3], home is also the place where people can best manage the consequences of their aging, which include illnesses like dementia [4] or physical decline [5, 6]. Furthermore, many older persons express strong reluctance to give up their familiar home for a nursing home, and often it is their next of kin that initiates the transition [7]. The reason for this attachment to one’s home is both practical, emotional and social [8]. Living in the same environment is likely to create a strong “embodied” experience [9], visual and other sensory impressions are deeply embedded in the memory and often shared with people who have occupied the same familiar space, therefore cementing the relationships, the personal narrative and the identity of a person [10]. Furthermore, giving up one’s home as an older person implies that she is no longer capable of living independently, which may be hard for many older persons to accept [7]. This dual aspect of being attached to one’s familiar environment, as well as the associated capacity of living independently in that familiar space is described in the theory of belonging and agency [11–14]. This theory emphasizes the meaningfulness of one’s home as a place where one feels attached, sheltered, and likes to belong (which is formed through experience), next to the more obvious aspect of functionality or agency one experiences in their housing (which is expressed through behavior) [13, 15]. To prevent moving away from their familiar home context, older persons may develop strategies to manage their deteriorating capabilities. This usually includes assimilative and accommodative coping strategies, which happens simultaneously, according to the two-process-model [16–18]. Assimilative coping refers to adapting their physical and social environment as such that goals still can be pursued, such as for example asking a family member to help with heavy items when grocery shopping and purchasing smaller, lighter baking moulds, so that the goal of baking a treat for friends and family every week can still be achieved. Accommodative coping refers to readjusting one’s ambitions in such a way that blocked goals become less desirable or important and usually entails eventually refraining from tasks that have become too difficult or exhausting, and relying more and more on the help and care from others [6, 16–18]. An example would be a shifted focus onto the burdens and risks of gardening from originally considering it a pleasant activity by an older person that becomes unable to attend to her garden on her own.

The question then arises what is it about the home that makes it so important to older persons and what aspects of the home are particularly valued by them. According to studies investigating the transition to home after hospital discharge for older persons, the home is associated with security, recovery and personal control [19], as well as familiar routines that the older persons yearned to reinstall and did not want to be interrupted [20]. According to Wahl and Oswald, it is precisely this dual process of creating a feeling of belonging through prolonged time and accumulated experiences in the same place, as well as creating a feeling of agency by having meaningful impact in that space due to one’s autonomous behaviour, that shapes the experience of home [14, 15]. The home therefore fulfils at least two crucial functions for older persons: it presents an extremely familiar, secure environment, where they know every step, every door handle, and every light switch with such precision that they can safely and easily manoeuvre through their home, even with declines in physical and cognitive capabilities [21, 22]^1^ and it gives them a space where they can experience privacy, being yielded from outside observation and judgement, and therefore can act and do as they please [23].

Engineers and researchers have recognized this wish of older persons to stay at home and are therefore constantly developing technologies that support aging in place [24]. Such technologies include, but are not limited to:

- Fall detection sensors for safety in floors, walls or wearables, as falls are the most common among older adults [25].
- Toilets with monitoring and cleaning capacities to both monitor their digestion and to help keep them clean [26].
- Sleep monitoring mattresses or wearables to detect changing sleep patterns or insomnia [27].
- Motion sensors to track behavioral patterns and detect decline in physical functioning [28].

What these technologies have in common is their centeredness around the user’s physical body and wellbeing [13]. While loneliness and lack of social interaction is also an important risk in old age and technologies are developed to provide older persons with social stimulation [29], this paper will focus on technologies that have more of monitoring, alerting and nudging functions, rather than social or entertainment functions. This is because our paper focuses on the embodied experience of the home, so investigating technologies focused on bodily monitoring will most likely have a stronger impact on that experience. Nevertheless, companion and social technologies may also alter the experience of home, for older persons and for occupants in general, but potentially in another way than that proposed in this paper.

Acceptance and adoption of such monitoring technologies at home remains low [30]. Current research suggests that the question of acceptance is neither centered around usability and usefulness [31, 32], nor around accessibility [33], as an accessible technology can still be rejected by the end-user. Thus, other reasons must explain why acceptance remains low, and one of them seems to be the lack of familiarity [34–36]. Complexity is another barrier found in research [37], as well as the felt invasion of privacy by monitoring technologies [38, 39]. Furthermore, a systematic review by Felber et al. [40] uncovered the most common ethical barriers to the uptake of technology in caregiving for older persons, which are concerns regarding privacy, autonomy, responsibility, the reluctance regarding artificial interactions versus human ones, trust, ageism and stigma. The concerns regarding privacy are understandable, as monitoring technologies constantly gather data, recognize their patterns and habits, detect minute changes in those patterns, and foresee possible problems to make predictions enabled by AI about the person’s capacities and the associated risks [41]. Therefore, not only do smart homes “know” the occupant through their data, a new kind of disembodied “presence” is introduced in the home.

If familiarity and privacy matter for the acceptance of smart home technologies (SHTs) for older persons, it is worth exploring why this is the case and how these aspects can be protected when introducing SHTs for them. Thus, in this paper, we will dive deeper into a theoretical exploration of why these aspects of familiarity and privacy at home are important, especially to older persons. We will then show how the introduction of SHTs alters this perception of familiarity and privacy at home, especially, older persons who are likely to not be familiar with technologies. More specifically, we propose the concept of Somatic Capability Assessment (SCA) to grasp the decision-making process of older persons at home and show how SHTs potentially impact that assessment, altering the experience of the familiar and private home. The goal of this paper is thus to propose a new take on the interaction of older persons and SHTs and to examine how they modify the embodied experience of the home, bringing more understanding to the issues of acceptance of SHTs for older persons.

Specifically, we will begin by the proposition of the somatic capability assessment (SCA), a concept inspired by the theory of somaesthetics [42] that grasps how heavily involved the body is in our daily decision-making process, especially at home, and particularly, for older persons. Then, we show how the smart home potentially challenges this embodied process, altering the familiar and private experience of home, gathering evidence from related literature and empirical studies. In short, we hope to shed more light on the current major challenge of designing adequate SHTs for older persons, in order to facilitate the future success of these technologies. Lastly, we will mention current efforts in the literature that have potential to disrupt the SCA of older persons the least, and at the same time propose that such approaches should take the concept of SCA into account.

## The somatic capability assessment and why we propose it: theoretical background

The success of any new technology depends on its acceptability and adoption by the expected end-user [43]. In the case of older end-users, they still play a mostly passive part in the design and development process and designers fail to consider their feedbacks [43–46]. Part of the reason may be that around one third of the world population aged 65 and older is not using the internet [47] and thus are not fully recognized as potential end-users yet. However, it is also wrong to assume that all older adults lag behind in the adoption of innovations. Old age in and of itself describes a long lifespan, usually starting from age 65, yet that can last 30 years or longer for many. Thus, both objective capabilities of an older person, as well as their self-perception will influence their openness to technology, and many older persons remain open [48]. In this paper, we acknowledge the problem of ageism and the harmful stereotype of seeing all older persons as late adopters of technology [46, 49]. We therefore want to state that this paper may best describe the population of the currently oldest old, thus describing people that are aged 80 or older and that often already experience some form of cognitive or physical decline, yet at the same time usually only have limited internet and technology skills [47, 50].

Additionally, aging has been perceived in computer science research as a problem that technology can solve [30]. Researchers sensitive to such ageist attitudes have challenged this narrative [23, 51]. Although Human Computer Interaction (HCI) research in relation with older persons is growing [52], the gaps between SHTs designers’ assumptions and older persons’ actual needs and desires remain [53]. Furthermore, Jovanovic et al. [53] note that, while the aging body poses challenges in and of itself when it comes to HCI, “the body exists within a social and material environment, which is still unaccounted for by Information and Communication Technology research for older people” [p. 1947]. This social and material environment that the older person wishes to age in is the home. It is therefore time to consider the home when developing and designing SHTs for an older person’s body.

A philosophical theory that takes into account both the body and its environment is the theory of somaesthetics conceptualized by Shusterman [42]. It is a combination of the ancient Greek word for “body” (soma) and the discipline of aesthetics, originally meaning sensory perception. The term “soma” goes beyond the body as usually understood in everyday language, meaning the body as perceptive and purposive entity, forming an organic, living whole with the mind and connected to its environment [54]. Aesthetics are understood in the theory as broader than the usual meaning of appreciation of art and beauty, encompassing perception in general, both within the body and outside of the body. It can be seen as a sub-category of the philosophical discipline of phenomenology, developed from the writings of Merleau-Ponty, who emphasized the inseparable connection of mind and body [55]. Somaesthetics differs from phenomenology in two important points, however. On the one hand, it emphasizes the body as an active part of sensory experience, rather than a passive vessel in which the mind perceives experiences. On the other hand, the theory is deeply rooted in pragmatism and interdisciplinarity, rather than just descriptive analysis, proposing three dimensions of somaesthetics, which are analytic, pragmatic, and practical [24]. The analytic dimension is descriptive and investigates bodily perceptions and how they shape our reality (thus being close to classic phenomenology), the pragmatic dimension is normative, searching to improve the experience of the body and thus the experience of life. Practical somaesthetics encompasses actual practices of the discipline (potentially based on the pragmatic dimension), such as yoga, for example [55]. The interdisciplinary theory emerged in the twentieth century as a response to the growing interest of the humanities and social sciences in the body as an important object of research, providing a unifying framework for how and why the body shapes our experiences of our inner and outer worlds, and as an applied theory of the good life to offer guidance in how to make the most of those experiences [54].

The theory of somaesthetics seems especially relevant in the context of older persons, as they have spent more time with and in their bodies on the one hand, thus having a more extended relationship with it, and on the other hand are dealing with their changing and aging body [56]. Thus, while the body plays a central role for all human beings, the body occupies an even more important role in one’s later life [57].

With the help of somaesthetics as an underlying theory, we can illustrate the body’s role in an older person’s daily life at home. In geriatric research, activities of daily living (ADLs) include tasks such as getting up from bed, taking a shower, preparing, and eating breakfast and so on [58, 59]. While it is undeniable that all of these actions involve the body, the crucial aspect introduced by somaesthetics is the realization that the body plays a decisive role if an how each task is executed [42]. We propose to name this process of decision-making with and through the body to perform a certain action somatic capability assessment (SCA).

Getting up from bed in the morning will be used as an illustrative example. Deciding to perform this action has a somatic component—we do get up because we feel capable of doing so. This feeling or knowing of being capable, however, is an evaluation in and of itself, with and through the body, and will cumulate in a judgement, of being capable or not capable of performing the action. For a young and healthy person, this somatic capability assessment (SCA) that takes place before getting up from bed may not even be noticeable on a conscious level, as there is no pain, discomfort, or weakness associated with the task, and the person judges herself capable of execution without thinking of that assessment. Once the body’s capabilities have diminished, however (such as due to lumbago, for example), the SCA will receive more conscious attention, as the back pain will influence the way in which the body will execute the task of getting up from bed. Pain is a good example of how our experience can both be sensory and emotional, involving both mind and body at the same time [60]. Furthermore, pain and discomfort often slow down our movements, and actions, to the point where normal timelines can feel rushed and more time for everyday activities is required [61]. Somaesthetics proposes that discomfort and pain in the body can be understood as an invitation for reflection [62]. This proposal is supported by medical research, suggesting that non-judgmental reflection on pain and its accompanying emotions can be beneficial for chronic pain patients [63].

The concept of SCA tries to capture these aspects of increased awareness, often triggered by noticing diminished capabilities (because of pain, discomfort or weakness), cognitive processing (which may need more time than in the case of a perfectly healthy and capable person) and emotional response caused by the awareness of diminished capabilities. All components together form the SCA that ultimately leads to the judgement of if and how a certain action is executed. Thus, the SCA—the self-assessment through and with the body—is the ultimate decision maker when it comes to personal action—the subjective, personal assessment of how we *feel* about a certain activity and whether we *feel* capable of executing it [64].

While older persons are often overlooked pertaining to scientific research on Information and Communication Technology [65], studies of the somatic experiences of the elderly population exist, including studies on the experience of pain [66–68] and frailty [57, 69]. Such studies are directly related to the concept of SCA, as both pain and frailty impact one’s SCA in a negative way. If an older person not only experiences constant joint pain but also tends to feel vertigo when sitting up, even getting up from bed can be a challenge. As a response, persons suffering from such limiting conditions will slow their action down, in order to accommodate these conditions [61]. In doing so, this same person with pain and vertigo will often find a certain way to enable her to get up from bed without a problem, maybe by allowing herself 5 min in a seated position before standing, or using the bed’s headboard to pull herself up. Thus she has successfully incorporated her home into her SCA, rendering her still capable thanks to a familiar environment.

The aforementioned example illustrates how the home plays a crucial role when it comes to the SCA of older persons. As mentioned in the introduction, the aspects of familiarity and privacy shape the home experience significantly. Having now introduced the SCA as a concept that shows how an (older) person executes activities of daily living at home, we postulate that the SCA explains why familiarity and privacy are crucial for an older person to live well at home. First of all, the home offers a secure background for which the different routines and strategies have been rehearsed over a long time to offer familiarity [70]. This aspect arises through time spent in a certain space. It is the space where, courtesy of years spent, older people are intimately familiar with and can navigate well in. Thus, in assessing her capabilities, the older person knows what features of her home she can rely on and subsequently incorporating these familiar features in her assessment. In theory, this familiarity can arise anywhere if enough time has passed for the person to adapt and know herself within this space. This implies that an older person can have the same experience of familiarity in a smart home [71] provided that he/she has had enough time to become acquainted to the smart home and feels comfortable in it. This implies that generations of new retirees will likely have less difficulty experiencing familiarity in a smart home, as they are more likely to adopt smart home technologies early on [72]. Indeed, gerontologists foresee that coming older generations will not have any problem in accepting assistive technology in their home. They see an increase in acceptance of these smart home technologies not only due to the fact that they increase the agency of the older person living in the smart home, but also suggest that, in the case of robots or social virtual assistants, for example, the continuous pleasant interactions with the device could foster a feeling of belonging, rendering the technologies part of the experience of home [15].

We will comment on this future possibility later on. For now, studies indicate that this aspect of familiarity is crucial for elderly persons, as a sudden move to an unfamiliar environment (like a nursing home) often goes hand in hand with rapidly deteriorating capabilities and quality of life [73–75]. In addition, merely the prospect of possibly changing environment may already produce anxiety in older persons [76]. Part of the reason for this correlation between a familiar environment and wellbeing may be the perception of increased control, agency and autonomy [11, 12, 70, 77], a relationship that we will investigate further in the next section.

The second important aspect of the home is privacy, as the older person can be assured that she is in her personal, intimate space, protected from outside observations, judgements or interferences [78]. Experiencing privacy is crucial for the older person’s SCA, as he/she does not need to care about how she executes a certain task, as long as she is still able to do so, thereby giving her freedom to be creative and still experience independence. This wish for privacy is especially relevant in intimate care tasks such as bathing and showering, and giving up that privacy when a person’s capabilities diminish to a point where assistance is needed is often experienced as difficult [79]. Furthermore, the feeling of privacy invasion extends to non-human presence, as sensors in the home have been shown to produce a feeling of surveillance for older study participants [80, 81].

Now coming to the smart home, the crucial novelty that it brings to the old home is its capacity to add a new stream of external information to very familiar (cognitive) processes. By gathering data through sensors, the smart home observes its occupants and acquires very intimate knowledge about them over time [82, 83]. The detailed knowledge of the smart home and the ensuing suggestions and warnings can generate conflicts while assessing a situation or executing a task, as the assessment of the smart home may differ from that of the older resident. Ideally, data will support the somatic experience of the end user, reaffirming her decisions. However, SHTs can also disrupt the habitual somatic experience of an end-user in their home, creating unfamiliarity or so-called “estrangement” [84, 85]. While this method can be deliberately used to foster engagement or even creativity [84, 85], accidental creations of estrangement without purpose, for example in situations where the evaluation of the smart home negates the end-user’s self-assessment, risks disturbing the symbiotic relationship they have with their homes. This danger of “wrong” or undesired feedback and/or suggestions by the smart home to the occupant is especially pertinent in the beginning. Not only are the algorithms of the smart home not yet adapted to the person living in the smart home environment (known as the “cold-start problem” [86, 87], thus increasing the chances of mismatches, the period is especially crucial for the uptake of a technology, as problems or errors encountered early will dampen the acceptance of the technology [71]. This is why it is especially important to ensure a very smooth, symbiotic introduction of SHTs in the home of older persons, and why special attention should be paid to their decision-making process, their habits and their capabilities.

## Literature supporting SCA

While SCA as a concept is newly proposed and has not yet been investigated empirically, we will draw from research from two related fields to support our theory. Firstly, research investigating users’ experiences with monitoring technologies confirm the hypothesis of a disrupted SCA when technology is introduced. A study by Lehrer et al. [88] noted that some users of wearables were disturbed by the knowledge they received about how little they moved, while others who were already very physically active chose to disregard the data provided by the wearable in moments where the somatic experience (like shortness of breath) was different from what the current level of activity should feel like. Studies have also reported a motivating aspect of such data feedback [89]. However, the data can also cause frustration, powerlessness and annoyance, according to other studies [88–90]. The constant stream of data, especially if it comes with alerts or warnings, is likely to create a feeling of unfamiliarity, in the sense that the person never knows when the smart home would “wake up” to “scold” her, especially if the person’s routines have been maintained and the somatic experience has not changed. For example, receiving constant feedback about one’s sleep performance could potentially induce feelings of either tiredness (if the data suggests that one has not slept well) or even a feeling of guilt and confusion (if the data suggests that one has slept very well, yet the person feels tired and unrested) [91]. Thus, the silent, unchanging presence of the home has transformed itself into a waking evaluator, removing the sense of familiarity and privacy that the person has experienced previously. In addition, similar anxieties related to unfamiliarity or estrangement can be caused by the fact that technologies have the pronounced characteristic to evolve from simple updates of a currently used system to whole new technologies [92]. Furthermore, research is even done on sensors detecting emotional states and, while the results can seem impressive, they are nevertheless still lacking accuracy [93], adding another layer of potential problems in acceptance of SHTs of older persons.

In short, all health data gathered by the smart home such as the amount of steps, gait, heart rate, sleeping pattern, frequency of bladder and bowel movements (in addition to their quality), and movement patterns (to detect wandering, for example) can all be reported back to the older person, adding a layer of information to his/her SCA that is unfamiliar to them. This may drive a wedge between the person’s somatic experience and the decisions about her capabilities, creating a constant sense of unfamiliarity [90, 91, 94]. In this sense then, SCA questions the assumption outlined above that simple and prolonged exposure to smart home technologies will cause increased feelings of agency and belonging for older persons [11]. We do not claim that the hypothesis is wrong. Rather, SCA adds nuance to the assumption, as well as to the theory of belonging and agency in general, with its emphasis on the somatic component of these two processes relevant for the feeling of home. For example, we assume that it is reasonable to believe that the potentially caused estrangement through the constant stream of data by the smart home technologies can disrupt the feelings of belonging. Belonging, according to Wahl and Oswald [11], is a process connected to experience as well as the identity of the person. The question of “who am I” gains specific importance in the context of home for older persons, as this is their most familiar space, the space where they feel to belong the most [12]. It is possible that smart home technologies can provoke alternative, and potentially negative answers to that question. If gait or digestion analysis from smart home technologies produces statements such as “the resident is walking 10% slower than last week” or “the resident is eating 15% more food than last month”, these could potentially be translated by the smart home resident into affirmations like “I am old and weak” or “I am a glutton”, affirmations that would not have formed in the resident’s mind if they only had their own body and SCA to assess their gait and food during the last week. Of course, the inverse is also possible, where assessments by the smart home technology can produce positive affirmations. Nevertheless, the fact remains that this new stream of data modifies the sense of belonging to one’s home, at least in the beginning, where the new presence and feedback of the technology needs to be incorporated into one’s daily life. The impact of smart technologies on one’s identity in the home remains to be explored in the future, for now we consider it sufficient to say that it is not entirely clear that assistive technology will automatically enhance feelings of belonging, in our view, even if they very well could. Further empirical studies using the theory of belonging and agency in the context of smart homes will be needed to show the effects of the technologies.

Secondly, the other component of the theory, agency, which describes the behavioral component of living at home, is also one where SCA can add a layer of detail, especially in the context of a technology-enhanced home [11]. As explained in the description of the concept, SCA can be understood as an embodied expression of agency. By tuning into the body and assessing if one is capable of undertaking an action, one evaluates one’s agency and ultimately decides to undertake that action or not. While it is true that technologies can increase agency of older persons by assisting them in certain tasks, the purpose of SCA is to explain that this increase in agency will only be welcomed as long as the embodied experience of agency is not disturbed. For example, let us imagine a smart bed that is able to shift its height and incline in order to facilitate getting into bed and getting up from bed for older persons. While this facilitates a basic ADL for older persons and increases their agency at home, a bed that would not leave any embodied agency to the older person, letting them decide if and when they want to use the smart bed features (perhaps because he/she experiences plenty of mornings where he/she does not want to rely on the assistance of the bed) may likely not be well received by the resident. In this sense then, we propose to add the concept of SCA as a concept to the current literature on the theory of belonging and agency, which could strengthen its explanatory force regarding the importance of the environment of older persons, specifically the home [11–13, 15, 95].

Secondly, research investigating the acceptance of smart home technologies for older persons, using acceptance theories, such as TAM [96] or UTAUT [97] have emphasized the issue of dissatisfaction with the smart home’s functioning as a potential barrier [98, 99], where dissatisfaction indicates that the use and performance of the SHTs is not happening as smoothly as expected. Furthermore, the constant stream of data, which is not only gathered, but also analyzed and compared to a baseline, can create a sense of not only being watched, but also of being evaluated and compared [100]. This will likely cause stress and a feeling of intrusion and surveillance. Such situations will reduce the end-user’s confidence to function independently in their own home. Interestingly, this confidence in functioning independently at home, even without technology, seems to increase acceptance of technology [98], and that lack of confidence in themselves in general makes older persons less likely to accept technology [34]. This indicates that, if the now “smart” home is likely to create feelings of doubt and low self-esteem in the older occupant, because of either dissatisfying functioning or evaluative processes that make the older person feel judged, than the acceptance of SHTs is likely to decrease even further.

The concept of SCA offers a potential explanation of the origins of both dissatisfaction and lowered self-esteem when technology is introduced. As the SCA emphasizes the “oneness” with the body and the mind’s reliance on it to make decisions about tasks and execute them, having to use the body to interact with SHTs, thus creating unfamiliar and potentially frustrating experiences will impact the SCA negatively. Indeed, research emphasizes that new technologies are often not built for older person’s bodies [34, 101], given that they often suffer from conditions such as decreased hearing [102] or trembling [103], which makes it harder to interact with voice-assisted devices and touchscreens. Having to deal with technologies that are built for younger, more functional bodies will most likely create feelings of frustration with one’s aged body, causing dissatisfaction and low self-esteem. Here again, we consider the SCA as a valuable addition to current theories of technology acceptance. Both TAM and UTAUT mostly rely on cognitive evaluations to judge technology acceptance, such as perceived usefulness or performance expectancy, or perceived ease of use or effort expectancy [96, 97]. While recent refinements of the theories pay more attention to affective and behavioral factors influencing acceptance, such as hedonic motivation or current habits [104], we are currently unaware of efforts incorporating embodied affective states into these models to evaluate acceptance. SCA with its emphasis on how one *feels* when interacting with the technology could add important insight to technology acceptance, potentially providing more understanding to the reasons why end-users enjoy new technologies or get frustrated by them.

The last and most apparent change that STHs bring to the home of an older person is the (felt) intrusion into their private space. Studies such as Epstein et al. [105] suggest that the presence of sensors, cameras, or other monitoring technologies create a feeling of being constantly watched, while Cannizzaro et al. [72] discovered that older persons are indeed more worried about privacy intrusions in smart homes than younger citizens. Constantly worrying about privacy intrusions and/or managing perceptions of being watched is disruptive to the embodied home experience and the SCA for two reasons. Firstly, the usual internal process is disrupted by the thought of the issue of privacy. As the home is recognized with the feeling of privacy, no cognitive effort was allocated to the question if this is actually the case or not by the resident. Once the perception of privacy is disturbed, the SCA would shift accordingly due to a likely amount of awareness of publicity. Second, this shift in the internal process would probably influence the external result of the SCA, whereby this manifests in bodily changes. A sensor in the ceiling or wall may cause the older person to change her usual spot of getting dressed and would pick a spot where she thinks the sensor would not be monitoring her images and/or movements.

## Design approaches related to the SCA and how SCA can enhance them further

As mentioned in the introduction, SHTs have the potential to benefit the health of older people in their homes and to facilitate aging in place. Thus, the support, guidance, and comfort they offer must be designed in a way that older end-users are willing to accept them. If they perceive too much unfamiliarity and/or fear privacy violations, they will likely reject SHTs [106]. As we have focused on the importance of the body and the somatic experience of older persons, we suggest design solutions that take this somatic, embodied experience into account through the SCA. Fortunately, some design approaches are already doing so, and mentioning them here will give more prominence to these efforts, as well as give more detail to interested researchers and designers.

Among the most prominent embodied theories, Shusterman [42] suggests including the body in architectural design in general, without going into the technological realm of smart architecture yet. He argues that the body is the general means through which we experience architecture and the only reference point we have in relation to concepts like distance, mass, symmetry, and so on. Furthermore, he proposes the discipline of “pragmatic somaesthetics”: the study of methods meant to improve the body as a tool of perception and consciousness. He concludes that bodily perception can hardly ever be separated from the environment in which it occurs. Therefore, the process of creating environments should take the body into account. Incidentally, putting the physical body at the center around the organization of the home is an approach that has recently gained interest due to the COVID-19 pandemic. This is because social distancing and remote working require the body to stay in a confined space (the home), and to integrate activities that commonly took place outside the home into the home, which was also feasible through technology [107, 108]. Furthermore, ICT scholars have noted the absence of the body as a research topic when it comes to developing and designing technology for older persons [53] and the importance of the context in which older persons use the technology [30].

One way to introduce the body into the design of SHTs for older persons is the possibility to use familiar appliances, sounds, and moving patterns for technology to create a familiar connection between the older person and the technology. This approach is called “tangible interaction” [109], and can be used both in a peripheral way, meaning that the interaction happens in an automated manner that uses less cognitive resources, or in a focused way that requires the full attention of the user. Easy examples of this would be the integration of familiar ringtones from old phones into new devices. A smarter home-related feature could be the use of familiar door and window handles. To refrain from wasting energy, smart homes may be designed with no or less windows that can be opened by the occupant [110]. However, to induce a familiar feeling, the window knobs and handles an older person was used to could be installed in the smart home, too, for example, open actual windows. For example, one could imagine a door or window in the bathroom or bedroom behind which embedded sensors measure body temperature, heart rate, and other physical markers. After getting up, the older person would stand in front of the familiar door or window, open it effortlessly (as he/she is familiar with the movement required to do so), and take their morning check-in. While the smart home is measuring the parameters, the screen could show a familiar scene to the older person. While it remains an open question how the recreation of an actual familiar scene, sound or scent will be able to instigate the same feeling of home than the actual experience of the familiar artefact, research at least indicates the importance of these familiar aspects for the experience of the overall home [70]. In incorporating such customizable features into the overall design of SHTs, the home experience is less disrupted when introducing SHTs into the home, thus increasing the acceptance of them by older persons.

As reintegration of familiar designs, sounds, appliances and more are not always possible in SHTs, another possibility is the creation of an established pattern and a familiar routine over time [94]. As an example, Höök co-created the invention called “Sarka”, which is a tool designed to encourage the user to produce very small, subtle movements, such as a minimal shrug of the shoulder or a soft balancing from side to side, while merely concentrating on how these movements are produced and felt in the body [94]. To foster this process, “Sarka” captures the movement through sensors, which are then translated into sounds. For an older person, “Sarka” could be incorporated into their mattress. As the sensors in the mattress are already meant to capture movement during sleep, the same sensors could be used for a meditative exercise (if desired by the resident), which could be done in the morning, the evening, or both. The routine could establish a sense of familiarity, making the smart mattress an integral part of the home. Furthermore, the exercise may foster a certain familiarity with the resident’s own body, through the meditation-like exercise. Studies have shown that meditation exercises can have positive effects on depression, pain management and cognitive abilities [111], which is also true for older practitioners [112]. Indeed, design inspired by somaesthetics is gaining some traction in different areas, such as mental health [113], pain regulation [114], female health [115] and fitness tracking [116]. Furthermore, researchers employing somaesthetics are trying to include the approach in the curriculum of technology design students, which then produced ideas for somaesthetic kitchen appliances in a workshop [117].

To summarize, such somatic design approaches, utilizing familiarity, either through things or designs that already hold familiarity for the resident, or through fostering familiarization with meditative exercises, seem the subtlest way in which technology could be helpful to a smart home resident, as they offer the smoothest incorporation approach into a person’s SCA. Taking the example from earlier about the SCA to get up from bed, the somatic mattress fosters awareness of one’s physical capabilities and limits, thus even possibly enhancing the SCA said person.

Both approaches mentioned above could also be used to design technologies which convey a feeling of respect and control of one’s privacy. In the example of the measuring window, closing the window with a turn of the handle would signify that the system is shut off, giving the user a sense of control over the technology. Or in the case of the mattress, a distinctive sound could indicate the shutting down of the system. Brightening or dimming of lights could also inform the resident about the status of a monitoring functions as well as vibrations in door handles, wearables, or appliances. In the somaesthetic designs produced by students mentioned above, smart kitchen appliances produced more radiant color the more they were used [117]. In the same manner, intensity of light could indicate how many users are currently looking at the older person’s data, for example. Similar to the way that owners of pets would always be marginally aware of the presence of their dog or cat by hearing them purring or yawning, or by feeling them when they approach closely enough to touch the resident, a person could become marginally aware that the smart home is “there” through such somatic markers. Intuitive actions, like flipping a switch, unplugging a device, or turning it away so that it does not face the user anymore, are all options that HCI designers could give for users to shut down technologies in order to preserve the feeling of privacy when wanted [109].

## Outlook

The examples mentioned above regarding the implementation of somaesthetics in design show how the theory is slowly making its way into HCI and technology design. Nevertheless, a next step is to properly incorporate the SCA in design cycles of smart home technologies. Thus, we mention several methods that would set the right fame to do so.

One of those methods is User-Sensitive Inclusive Design (USID). It belongs to the empathy-based design approaches [118], which emphasizes direct interactions and connections with concrete future users [119]. As humans age differently and consequently develop different needs, this produces special challenges in designing technological solutions to support their aging [120]. However, older users are usually simply compared to younger users, who are considered as “normal”, which carries with it a normative connotation [121], or are otherwise standardized to create “representative older users” [119]. USID however takes into account the individuality of each user and promotes an empathic process to find the right design solutions, working with or even starting from special, unique potential users [119]. Taking this a step further, the model of Responsive inclusive Design (RiD) [33] suggests to incorporate the evolution of the user into the designing process. Proposed as a way to ensure that people with disabilities receive appropriate devices for their needs, this approach takes into account that the needs and capabilities of end-users change over time. Thus, these end-users need to be regularly consulted in the designing process, and the designers need to be responsive to their suggestions. As aging represents a time where needs and capabilities of end-users change as well, RiD may be a promising approach for the appropriate design of SHTs for older persons, especially if older end-users are consulted about their SCA when using the device, ensuring that the interaction becomes as symbiotic as possible.

Another possible approach is the Value Sensitive Design (VSD), which acknowledges how value-laden and impactful design choices are [122]. For example, technologies promoting independence (a standard of normalcy with a normative aspect) rather than interdependence [123] make a clear choice when it comes to values. Soma designs and tangible interaction approaches could assist in identifying pertinent values and potential conflicts [124], as they emphasize how a certain activity or use of a product feels for the user through their own somatic experience, thus taking into account their individual experience and value system. In fact, researchers using the somaesthetic approach recognize that the values of end-users relate to their “human needs, desires, fears and hopes” [125] [p. 271] and thus propose to take the end-user’s values into account when designing a technology, in order to facilitate an interaction between human and device that is the most satisfying for the end-user. Somaesthetics and VSD therefore could be combined into a new design approach that recognizes how the embodied experience of end-users shape their interactions and thus acceptance of a technology, giving them a possibility to voice what they value when using a certain device. Indeed, researchers are already employing VSD as an approach to design smart home technologies, and the values they are suggesting to incorporate are similar to the concept of SCA. For example, Umbrello investigates how meaningful human control (MHC) can be incorporated into the design of a smart home assistant, with the goal of ensuring that decisions made by the assistant (such as certain grocery purchases or energy consumption decisions) are in alignment with the resident’s feeling of authority over the system, thus prioritizing autonomy and privacy of the resident over potential optimizing of automated decision-making happening in the background, in order to not create surprises that may feel intrusive or may cause feelings of estrangement in one’s home [126]. In the design of robots for caring purposes, which includes older persons as potential users, a modified VSD approach is proposed by some scholars, where human vulnerability should be incorporated as a value and where the focus of robotic design should lie on an embodied presence (rather than a small, wearable device or an unobtrusive voice-only presence) and interaction with a being (rather than tool-like assistance, to which no quality of being is ascribed), both with the purpose of fostering embodied awareness of the user when employing the robot for their care needs, similar to what the SCA proposes [127].

The SCA could specifically be introduced in design protocols, asking older persons how they evaluate and execute certain ADLs at home without any assistance, how and what they feel while doing so, why it is important to them to still execute the ADL themselves and how they wish to be assisted in the process by technology. This would provide approaches like VSD, RiD and USID with direct inputs from older end-users regarding their needs, desires and values, creating SHTs that would ultimately be perceived as part of the “home” by the older resident.

## Conclusion

This paper started by describing the importance of home for older persons, as well as what specific features of the home are relevant for them, which are familiarity and privacy. While SHTs are meant to support older persons at home, they can also seem unfamiliar and intrusive, especially as older persons are not used to rely on external assessments relevant for managing their daily lives and assessing their capabilities, especially at home. This creates a possible mismatch between SHTs’ intended purposes and older users’ needs and desires, complicating acceptance and adoption of SHTs. We proposed and used the concept of SCA to describe this problem and to grasp this idea of reliance on one’s own bodily perceptions and experiences, inspired by somaesthetics [42] and supported by related empirical research. Subsequently, we have explained how the introduction of SHTs is likely to alter the sense of familiarity and privacy of a person in her home, thus adding a problematic layer to the introduction of SHTs for older persons. Finally, we presented HCI design solutions that, in our view, already take the concept of SCA into account to a certain extent and could mitigate the aforementioned disturbances, helping to maintain the feeling of “home” for older persons and enabling a successful aging in place with the assistance of SHTs. In short, our goal was to advance and enrich the discussion around the ethical and effective design and implementation of SHTs for older persons.

## Footnotes

1 The first author has observed that her great-aunt, aged over 90 and having lived over 30 years in her apartment, moved flawlessly through her home despite having gone blind. She had achieved complete symbiosis with her home.
